# Advances in Pharmacological Treatments for Cystinosis: Cysteamine and Its Alternatives

**DOI:** 10.1021/acsptsci.5c00633

**Published:** 2026-01-15

**Authors:** Aitor Carneiro, D. Heulyn Jones

**Affiliations:** † Medicines Discovery Institute, School of Biosciences, 2112 Cardiff University, Main Building, Park Place, Cardiff CF10 3AT, U.K.; ‡ School of Chemistry, 2112 Cardiff University, Main Building, Park Place, Cardiff CF10 3AT, U.K.

**Keywords:** cysteamine, cystine, cystinosin, cystinosis, lysosomes, rare diseases

## Abstract

Cystinosis is an inherited lysosomal storage disorder characterized by the intralysosomal accumulation of crystals of cystine. This alteration is caused by the absence of the lysosomal membrane transporter cystinosin, which leads to clinical manifestations of the disease. Oral administration of aminothiol cysteamine, while not a curative therapy, has proven to be effective in controlling the progress of the disease and reducing its complications. However, the numerous side effects inherent to the treatment are responsible for low patient compliance, severely impacting therapy success. Several studies have been performed in the past few years with the aim of optimizing cysteamine therapy to avoid its main drawbacks. This review focuses on the potential and feasibility of these novel strategies. As well, it introduces novel recent approaches studied as an alternative or complement to cysteamine treatment.

## Graphical abstract

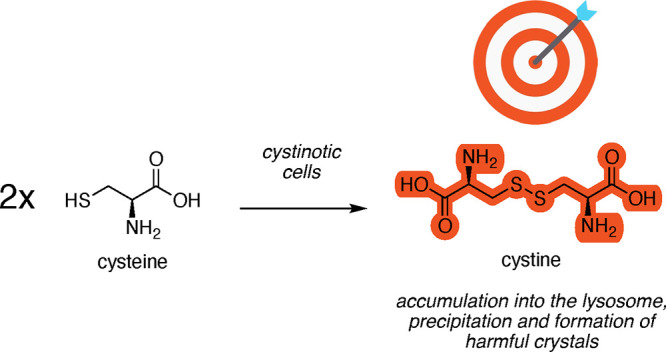

Lysosomes are ubiquitous intracellular organelles that were first discovered in 1955 by De Duve et al.^1^ The main differential characteristic of these important organelles, crucially involved in eukaryotic cell clearance, is their acidic internal pH, where the hydrolytic enzymes present at their lumen are optimally active.^2–4^ Understanding of the lysosome has greatly evolved since its first description, leading to a wider conception of it that comprises both the lysosome itself and its linked constituent parts in the cell, which globally act as an integrated system known as the greater lysosomal system. This system plays important roles in signaling, immune recognition, or macromolecule degradation and recycling. The molecular degradation and recycling role that lysosomes play in cells to maintain the physiological homeostasis are in fact determined by its links with the ubiquitin-proteasomal and autophagosomal systems. To maintain this whole system (Figure 1), lysosomal membrane proteins are essential, as they allow the salvage process to remove the degradation products generated after lysosomal processing, while some of them (e.g., TRPML1) are also involved in the regulation of different processes modulated by signaling mechanisms from the lysosome, such as autophagy.^2,5,6^

**Figure 1:**
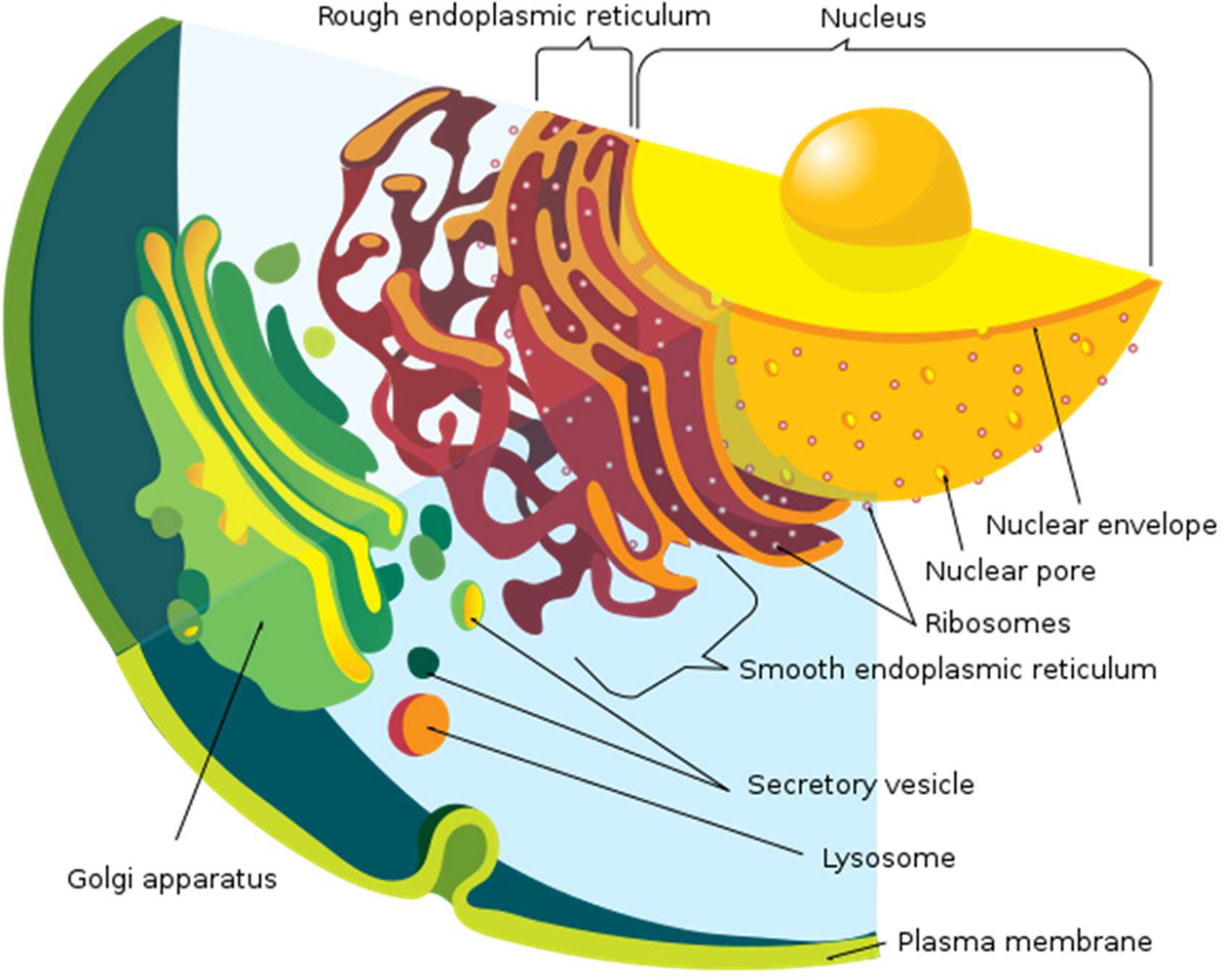
Diagram showing the situation of lysosomes in the endomembrane system. Illustration available in the public domain through Wikimedia Commons, ref 7. Credit to Ruiz.^7^

When deficiencies in certain key proteins occur, lysosomal diseases arise. These deficiencies often, but not uniquely affect lysosomal hydrolases; there are multiple targets both lysosomal and nonlysosomal that are critical for the proper function of the greater lysosomal system.^8^ Lysosomal storage disorders (LSDs), which were first defined in 1963 by Hers et al.^9^ simply as lysosomal enzyme deficiency states, can indeed be caused by dysfunctions at different parts of the greater lysosomal system. These disorders are mainly characterized by the progressive accumulation of endogenous macromolecules due to these dysfunctions, which are inherited and based on a genetic defect.^4^ While LSDs are usually multisystemic,^3^ they tend to have an important impact in the brain, as neurons seem to be particularly affected by lysosomal failure.^5^ Most disorders present at a young age, usually affecting children, although adults may be underdiagnosed. At present, close to 60 monogenic disorders are known. While it can significantly vary depending on geography, their combined frequency is approximately 1:5000–7000 live births. The severity of these pathologies, which are commonly related to childhood neurodegeneration, explains the growing interest in the field.^2,10^

Although clinical classifications are often accurate, the complexity of the mechanisms that regulate the greater lysosomal system activity can mean that definitive classification of LSDs is challenging, as many pathological features or storage material overlap across different diseases. The traditional biochemical classification is based on storage products and can be used as an initial guide, e.g., lipidoses, glycoproteinoses, mucopolysaccharidoses, and glycogen storage diseases. However, a more advanced classification based on the nature of the molecular defect in the greater lysosomal system is needed to differentiate groups of diseases with different storage products that share clinical and pathological features, and especially to classify certain diseases that cannot be fit into the former traditional classification, such as cystinosis, where the alteration occurs at the lysosomal membrane.^11^ In line with these observations, this review will be specifically focused on the biochemistry and molecular basis of the condition and will detail the medicinal chemistry strategies that have been attempted to tackle the disease in relation to its chemical pharmacology.

## Cystinosis

Cystinosis is an inherited autosomal recessive disorder affecting a lysosomal membrane transporter in which CTNS, a gene located on chromosome 17q13 that encodes for cystinosin, a H^+^-driven transporter of cystine, the dimer of the amino acid cysteine (Scheme 1),^12^ is altered. This genetic alteration, which is most commonly a large deletion,^13^ results in intralysosomal storage of cystine crystals that is predominantly responsible for the symptoms of cystinosis. Existing sources estimate that cystinosis has an incidence of 1 in 100,000 to 200,000 newborns, affecting around 2,000 patients worldwide.^14–16^

**Scheme 1:**
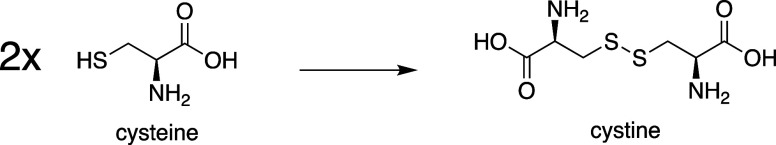
Dimerization of the Amino Acid Cysteine to Form Cystine

While other clinical presentations of cystinosis have been reported, children are the main age group affected, with life expectancy and quality of life severely impacted. The most common presentation of cystinosis, which causes an infantile or nephropathic disease affecting 95% of patients, is detected several months after birth in the form of Fanconi renal tubulopathy. This syndrome is caused by the failure of renal tubules to reabsorb small molecules, and the symptoms observed include vomiting, dehydration, electrolyte imbalances, or a failure to grow.^14,17^

Although kidney involvement is the most characteristic manifestation of the disease, due to its increased sensitivity to ischemic and toxic insults,^15^ a continued accumulation of crystals of cystine in lysosomes can also result in ocular, gastrointestinal, pancreatic, neurologic, or, most commonly, pulmonary affectation.^17–19^ For this reason, renal transplant cannot be considered a definitive solution for patients, although required in many cases to avoid death due to renal failure, and the development of less invasive and more effective treatments has been the principal goal of the research made in the area. At present, patients have a life expectancy beyond 50 years when treated.^20^

Research performed over the past decades indicate that the lack of cystinosin not only leads to the accumulation and subsequent formation of crystals of cystine in the lysosome but is also responsible for reduced cellular energy metabolism, oxidative stress leading to mitophagy, increased apoptosis, defective mTOR signaling, abnormal autophagy, and inflammasome activation.^21^ These additional phenotypes may depend on the cell lines that were studied. For an in-depth discussion on the native structure and mechanism of human cystinosin and how those connect to the consequences observed for its alteration, we recommend consulting the recent study by Guo et al.,^22^ where the structures of human cystinosin in lumen-open, cytosol-open, and cystine-bound states are reported, uncovering the cystine recognition mechanism and capturing the key conformational states of the transport cycle.

After describing the basis of cystinosis and its treatment, this review covers the recent advancements in new pharmacological treatments and discusses their molecular mechanisms, highlighting their potential advantages and disadvantages.

## Pharmacological Treatments for Cystinosis

### Cysteamine

As previously mentioned, the deficiency in the transporter cystinosin leads to the accumulation of cystine, which crystallizes inside the lysosome. Therefore, an intuitive option from a chemical point of view can be targeting cystine with a compound capable of performing a nucleophilic attack on the disulfide bridge to release cysteine, which is soluble under physiological conditions and is able to exit the lysosome. This strategy is indeed the one followed by the available treatment: the oral administration of the aminothiol cysteamine.^23^ This widely used drug works by entering the lysosome via a specific transporter and depleting dimers of cystine, producing cysteine and a mixed disulfide which are able to leave the lysosome using a lysine carrier, which is a cationic amino acid transporter (Scheme 2A).^24,25^ Besides its use in cystinosis, cysteamine has been reported to have potential therapeutic value in other disorders, such as Batten disease (another LSD),^26^ Huntington′s disease, and nonalcoholic fatty liver disease.^27^

**Scheme 2:**
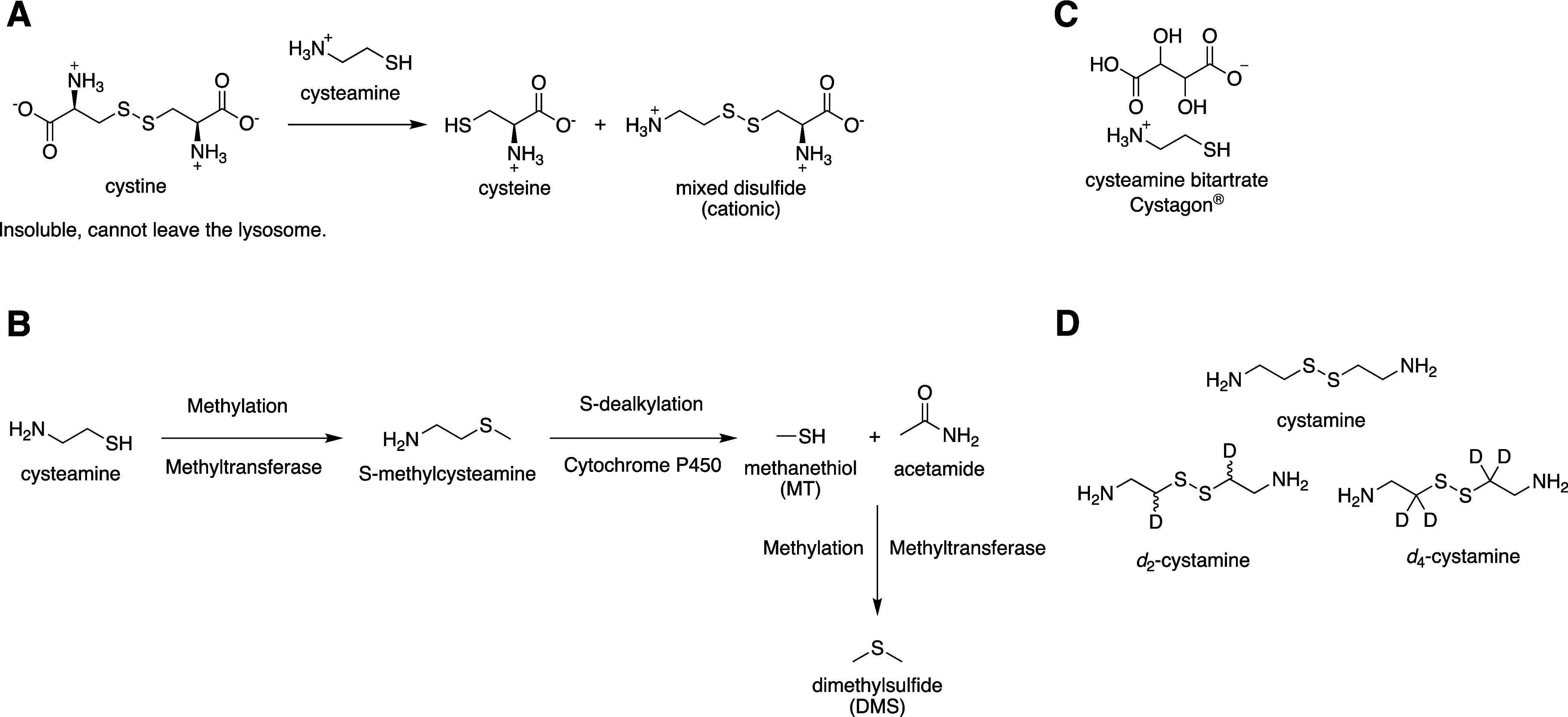
(A) Mechanism of Action of Cysteamine; (B) Cysteamine’s Metabolism Pathway, Which Leads to the Formation of MT and DMS, Two Volatile Metabolites Responsible for Halitosis and Body Odor;^29,32,37^ (C) Cysteamine Bitartrate Salt, Commercialized as Cystagon 150 mg Hard Capsules; and (D) Cystamine and its Deuterated Derivatives, Reported by Leszczynska et al.^36^

It must be considered that cysteamine is not a curative treatment for cystinosis,^28^ as it cannot replace cystinosin, and data reported by some studies suggest that it fails to completely clear cystine storage in tissues.^17,29^ However, cysteamine has proven to be an effective therapy for improving the quality of life of patients and increasing their life expectancy significantly. The progression of renal complications (Figure 2) and damage to extra-renal organs is slowed. The effect of cysteamine is short-lived, mainly due to its extensive first-pass metabolism (Scheme 2B), which reduces its bioavailability to an estimated 10–30%.^30^ The result of this is an administration guideline of 4 daily intakes (6 h dosing interval) that may disrupt sleep. Patient compliance is also negatively affected due to cysteamine’s unpleasant odor and taste, exacerbated by its ability to bind to oral mucosa and dental fillings. Thus, it is preferable to administrate it as a bitartrate salt (Scheme 2C). Nevertheless, even in that presentation, cysteamine causes nausea and vomiting in several patients.^31^ Other unpleasant side effects include gastric or duodenal ulceration,^32^ halitosis, and body odor caused by its volatile metabolites.^14,33^

**Figure 2:**
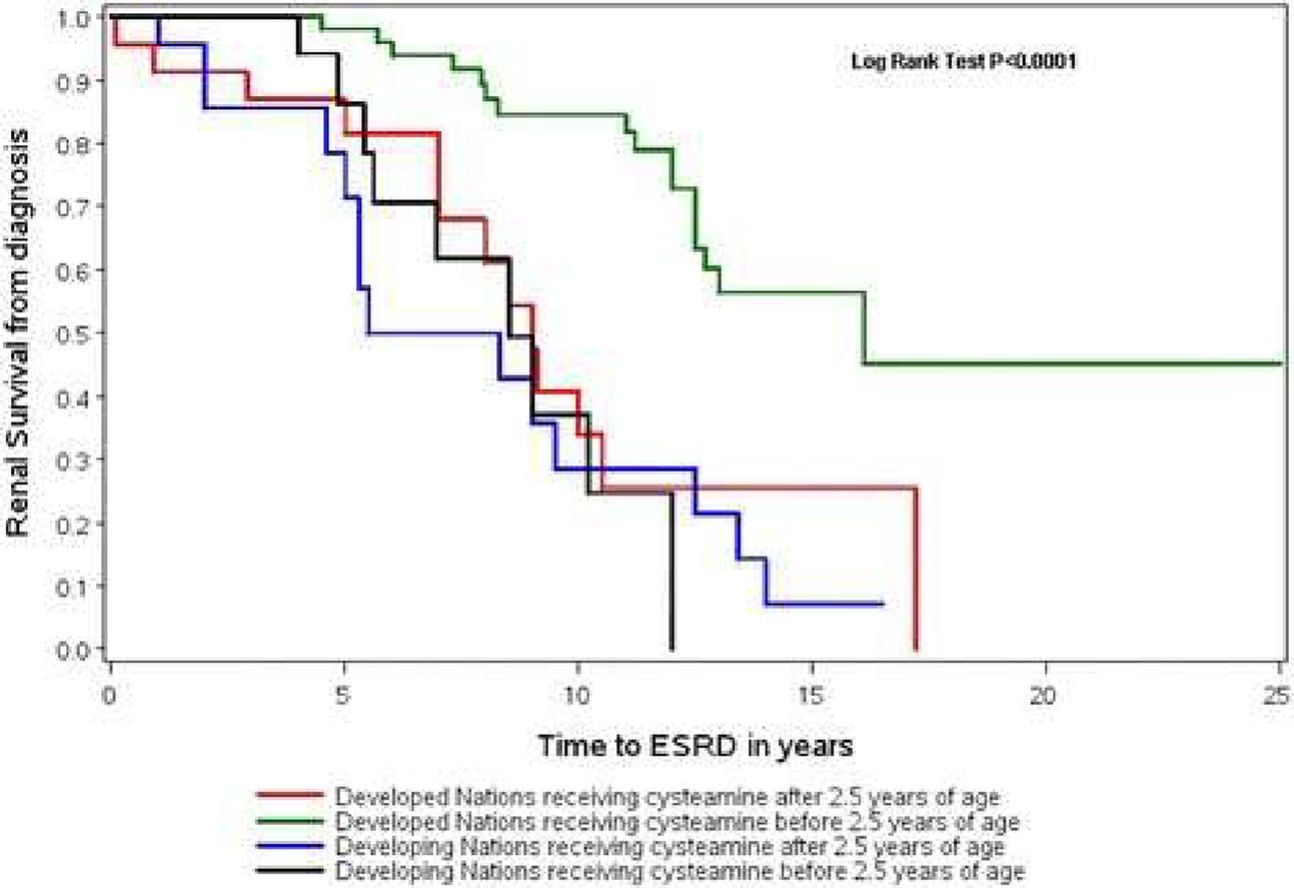
Comparison by Bertholet–Thomas et al.^34^ of time to end-stage renal disease (ESRD) in patients from different countries depending on age initiation of cysteamine treatment. Data on Developing Nations did not show statistical differences, which can be partly explained by the difficulties in accessing adjunctive measures. Reprinted under a CC BY 4.0 license from ref 34. No changes were made.

Despite the success of cysteamine in delaying disease progression, the drawbacks and side effects of this drug have been the cause of low compliance in patients, especially when they reach puberty. A study by Ariceta et al.^35^ estimates a self-reported treatment adherence of 89% in Spanish patients under 11, while this number is severely reduced to 56% in older patients, which are much more commonly responsible for their own treatment. Consequently, those patients can suffer a more rapid degradation in their renal function and are more likely to see extra-renal complications of disease progression. It is of vital importance, therefore, to develop solutions to the major causes of this low compliance.

The main efforts made have been directed toward two different but complementary approaches: the development of sustained or delayed-release cysteamine formulations to control the liberation of the drug without modifying its chemical structure and the design of prodrugs that may protect cysteamine until it reaches its target. In addition to these, a promising recent study by Leszczynska et al.^36^ points toward the potential of deuterated cystamine derivatives to reduce the rate of formation of the discussed volatile sulfur metabolites, although pharmacokinetic studies are yet to be performed to fully confirm this hypothesis. These deuterated cystamine derivatives, dimers of cysteamine (Scheme 2D), have already proven their value as superior agents with respect to the outcomes observed in fibrosis, inflammation, and lipid peroxidation.

### Cysteamine Delayed-Release Formulations

An initial approach to solve the problems of classic oral cysteamine administration was the modification of its pharmaceutical formulation to develop the delayed-release form Procysbi, which allows dosing at 12 h intervals. This prolongated interval is achieved by incorporating an enteric coat to the capsules,^38^ an idea that was based on a study performed in 2007 by Fidler et al.,^39^ which concluded that direct administration of cysteamine into the small intestine led to higher plasma levels when compared with gastric administration. This larger dosing interval alleviates sleep disturbance, but it does not provide any solution to the gastrointestinal issues observed, as cysteamine continues to be released in the gastrointestinal tract. This gastrointestinal tract release does not address another major issue of cysteamine treatment: the intense first pass metabolism, which leads to low bioavailability and the formation of strong-smelling volatile metabolites MT and DMS.^23,40^ There is, however, a study performed by Besouw et al.,^41^ which suggests that the levels of DMS in expired air might be lower after administration of the delayed-release formulation, while cysteamine area under the curve (AUC) remains the same, which can indicate that the objective of increasing bioavailability relative to current treatments releasing the drug directly into the small intestine could not be achieved.

Although there is a recent study performed by Berends et al.^42^ investigating the possibility that a novel sustained-release cysteamine bitartrate formulation (PO-001), which uses a non pH-dependent coat,^43^ could lower the peak levels of cysteamine, thus reducing halitosis issues, the development of these sustained-release formulations does not seem to be a definitive solution.^28^ Simply improving the formulation of cysteamine leaves many of the problems observed in the treatment of the disease unsolved and cannot progress toward an actual curative remedy.

### Cysteamine Prodrugs

When Anderson et al.^44^ first pondered the possibility of designing cysteamine prodrugs, it was highlighted in their study the importance of both amino and thiol groups in cysteamine. By analyzing molecular modeling studies comparing the shape, size, and relative position of functional groups between lysine and cysteine-cysteamine dimer, it was stated as a conclusion that both functional groups are crucial for the activity of the drug. While the thiol group allows the formation of the mixed disulfide with cysteine, the presence of an amino group in a β-position to it is also required in order to retain a sufficiently similar structure to lysine to ensure exit from the lysosome.

Considering the findings exposed above, it was undesirable to completely change the structure of the drug, but the possibility of modifying its functional groups with prodrug moieties that could be cleaved once the compound reaches either systemic circulation or the cell (depending on the design) stayed open. This prodrug strategy had the potential to minimize many of the drawbacks of conventional cysteamine treatment, such as odor problems or gastrointestinal side effects. For this initial study, Anderson et al.^44^ proposed the conjugation of cysteamine with different α-amino acid structures. γ-Glutamyl transpeptidase was targeted by synthesizing γ-glutamyl-derivatives of cysteamine (Scheme 3A) with the aim of increasing uptake of cysteamine into the kidney, where this extracellular enzyme is highly expressed.

**Scheme 3:**
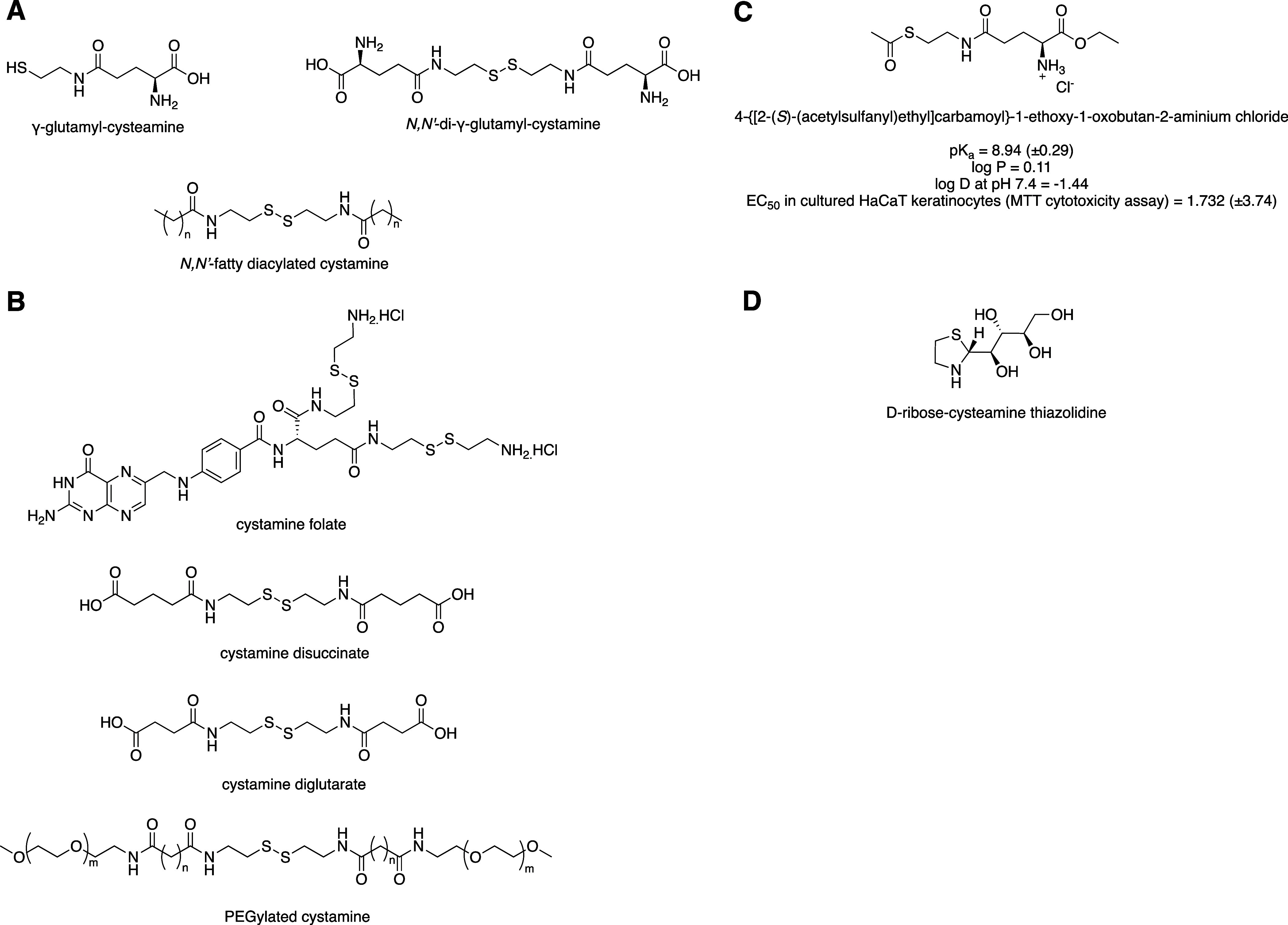
(A) Prodrugs Reported by Anderson et al*.* ^44^ and McCaughan et al.;^46^ (B) Folate, Glutarate, Succinate, and PEGylated Derivatives of Cystamine;^47–49^ (C) In Vitro Properties of an Example of Ester Derivative Synthesized by Frost et al.;^23^ and (D) Example of Carbohydrate-cysteamine Thiazolidine Synthesized by Ramazani et al*.* ^50^ in Their Study

Although the study showed interesting in vitro results regarding cystine-depleting ability and low toxicity against Chinese hamster ovary cell lines, this cysteamine-amino acid conjugation strategy had an important drawback, as the known rapid hydrolysis of amino acid prodrugs in blood would release cysteamine and fail to prevent its metabolism and formation of MT and DMS.^45^ Due to this reason, the synthesized prodrugs failed to show a definitive improvement to support their use in the clinic.^23^ Despite this setback, this initial study performed by Anderson et al.^44^ was followed by a new prodrug strategy by McCaughan et al.^46^ based on *N*-fatty acylated cystamines (Scheme 3A), which were proposed due to their capacity to improve the oral palatability of cysteamine. Among them, the decanoate derivative was selected, mainly for its favorable solubility in ethanol, to be evaluated *in vitro*. This analogue showed statistically significant lysosomal cystine-depleting capacity, as evidenced by HPLC determination of the levels of cysteine per quantity of protein in cystinotic fibroblasts upon 72 h of treatment with either the analogue or a control. These encouraging data was coupled with negligible observed cellular toxicity in alamar blue proliferation assays.

Despite not achieving success in progressing to the clinic, these two studies by Anderson et al.^44^ and McCaughan et al.^46^ were key in highlighting the potential of using prodrug approaches for developing treatments for cystinosis. In the following years, other cysteamine prodrug studies were performed, e.g., folate, glutarate, succinate, and PEGylated derivatives of cystamine (Scheme 3B), which also showed interesting in vitro results, particularly in terms of cystine depletion, whose relative concentration per mg of protein were significantly lowered in many cases in cells treated with these prodrugs when compared with untreated and cysteamine-treated cells.^47–49^

The study performed by Frost et al.^23^ must be highlighted, as it built on the work of Anderson et al.^44^ by synthesizing ester/thioester derivatives of the γ-glutamyl derivatives of cysteamine (Scheme 3C). These new derivatives proved in vitro to be more metabolically stable and to have adequate physicochemical properties for oral administration, as noted in Scheme 3C. In addition, they were able to maintain the concentration of cysteamine above baseline levels for at least 24 h. However, the results reported in this study have again yet to be confirmed by further in vivo studies in animal models.

Ramazani et al.^50^ studied carbohydrate-cysteamine thiazolidines (Scheme 3D) as alternative potential prodrugs for the treatment of cystinosis. In this case, it was concluded that the derivatives obtained, while promising, were too stable and needed to undergo further structural modifications to allow in situ release of cysteamine by intracellular hydrolysis.

As exposed in this section, there is a recent trend of studies in the literature progressing toward the development of clinically relevant cysteamine prodrugs. This is consistent with the hypothesized advantages of protecting cysteamine with different prodrug moieties to avoid its release before reaching the cell and, particularly, the lysosome, which leads to its typical side effects. However, it must be noted that most current studies have limited evidence from a narrow variety of chemical modifications, with data obtained from in vitro experiments only. Therefore, there is still great growing potential in this area, both in the development of in vivo studies to confirm the results already obtained and in the design of novel prodrug structures that could further optimize cysteamine effectiveness. Finally, it is important to mention that a cysteamine prodrug encoded as CF10 developed by University of Sunderland, which was initially funded in 2018 by the Medical Research Council with a grant to complete its preclinical development stage, has recently been awarded a second round of funding, securing a multimillion-pound investment to enter clinical trials in the United Kingdom.^51^ This news represents great achievement for the field.

To visually summarize this section on efforts toward cysteamine-based drugs with improved pharmacokinetic properties to reduce side effects of the treatment, a timeline highlighting the key advances throughout the last two decades is shown in Figure 3.

**Figure 3:**
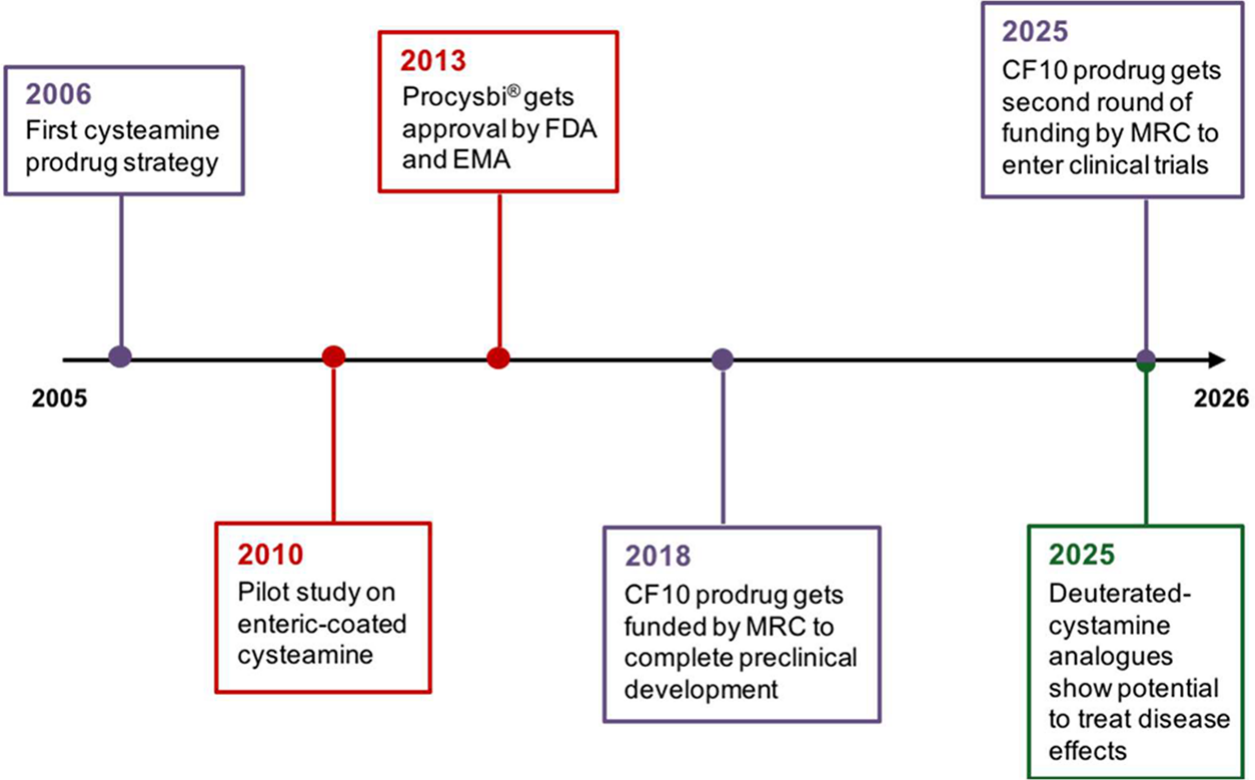
Timeline for the development of cysteamine-based drugs. In purple are shown the prodrug approaches. In red are shown delayed-release formulations. In green, there are deuteration strategies.

### Emerging Therapies for Novel Molecular Targets

Beyond the accumulation of cystine in the lysosomes, there are multiple pathways that are altered by the lack of regular cystinosin. Although a deep discussion of the biology underlying these alterations is beyond the scope of this review (see Jamalpoor et al.^21^), it is relevant to discuss the approaches made in the past few years regarding the discovery of new drugs targeting molecular targets which are thought to be involved in the disease. These novel therapies are mainly focused on treating nephropathic cystinosis, the most severe form of the disease.^52^ These are summarized and organized based on their mechanism of action below (Table 1).

**Table 1:**
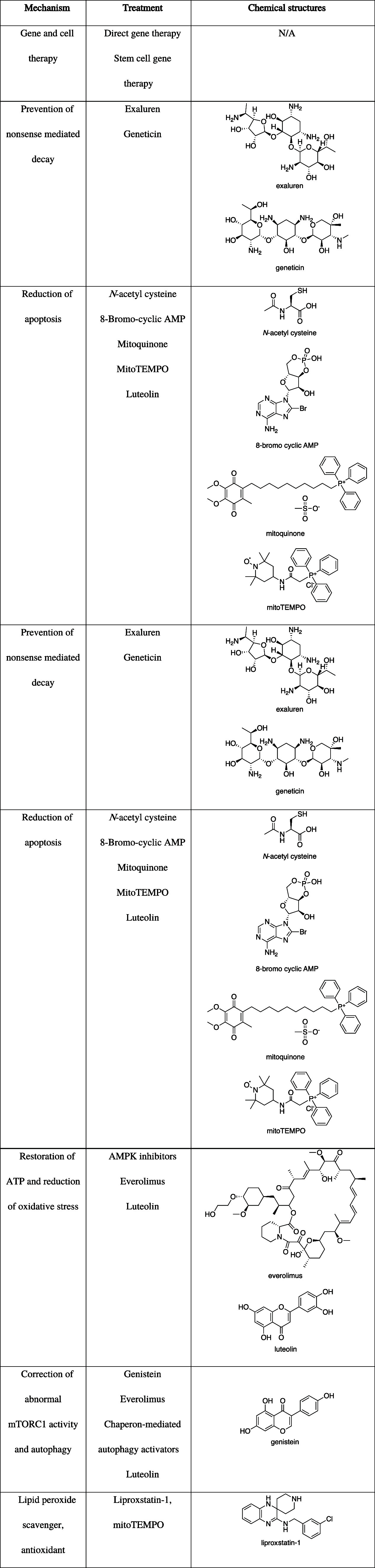
Classification of Emerging Therapies for Cystinosis with Mechanisms Not Involving Cystine-Depletion^a^

a Based on Jamalpoor et al.'s^21^ work.

Among the therapies listed, the relevance of everolimus, an mTOR inhibitor that performs many of the corrective mechanisms shown above while not affecting cystine load, must be highlighted. The defective mTOR signaling in cystinotic cells, which is believed to be related to the absence of cystinosin, leads to an abnormal activity that cannot be corrected by cysteamine, as the entire autophagy–lysosome system is disrupted with a potential blockage in autophagic flux after the fusion of autophagosomes with cystinotic lysosomes.^53^ In this context, everolimus is able to target a larger number of altered pathways in the disease involved in the so-called cystinotic phenotype that are not modulated during cysteamine-only treatment, for instance, by reducing enlarged lysosomes to near-normal levels via autophagy or reducing the levels of apoptosis. Based on this, Hollywood et al.^54^ proposed the study of a dual therapy combining the cystine-depleting effect of cysteamine with everolimus, which would give a more holistic approach to treat cystinosis. It was indeed concluded that both the restoration of basal autophagy flux and the reduction of apoptosis by everolimus were still observed in a dual treatment with cysteamine. Nevertheless, it must be noted that the corrective effects on cystinosis observed for everolimus are generic (i.e., not specific to cystinotic cells) and qualitatively assessed in the context of the disease.

Another promising molecule shown in Table 1 is luteolin, a natural flavonoid that is also able to target many of the different processes altered in cystinotic cells. Uniquely, this molecule acts on two unspecific altered processes in the cell that can be crucially relevant to the development of the complications of the disease: the reduced levels of intracellular ATP, which is believed to be caused by a reduced reabsorption of inorganic phosphate,^55^ and the increased oxidative stress, which may be influenced by an impairment on glutathione synthesis in cystinotic cells affecting the scavenging of reactive oxygen species.^56–59^ Both processes are thought to be closely related to the observed increased apoptosis rates and mitophagy.^21,60,61^ A recent study performed by De Leo et al.^52^ also emphasizes the interesting pharmacological profile of luteolin, which has already proven to show a good safety profile in humans.^62,63^ Specifically, luteolin was identified in a high-throughput screening on the basis of an in-cell ELISA assay for its capacity to reduce the levels of the autophagy-related protein p62/SQSTM1 in cystinotic cells, which along with its general antioxidant and antiapoptotic properties made it a suitable multifunctional agent to consider in cystinosis treatment. Given the inconclusive data regarding the ability of cysteamine to affect the mentioned altered processes,^53^ it becomes increasingly important to focus on the discovery of molecules showing broad pharmacological profiles, as luteolin does.

A recently discussed additional mechanism that explains the observed kidney damage in cystinosis patients is the abnormal metabolism present in podocytes.^64^ This leads to increased mitochondrial oxidative stress, which, in turn, results in lipid peroxidation and cell damage. Liproxstatin-1 (Table 1), an inhibitor of lipid peroxidation discovered during a library screen of 40,000 compounds by Friedmann Angeli et al.,^65^ has been shown to improve patient-derived podocyte function to a greater degree than cysteamine. The in vivo murine DMPK properties of Liproxstatin-1 have been measured, with encouraging half-life and oral bioavailability.^66^ A second-generation analogue, Liproxstatin-2 has been reported and evaluated, although the chemical structure is yet to be disclosed.^67^

As a more general approach to cystinosis, translational read-through-inducing drugs (TRIDs), which are being studied for the treatment of genetic diseases,^68^ may benefit from an adjuvant use of nonsense-mediated mRNA decay (NMD) inhibitors, which would maintain significant mRNA available for the action of TRIDs, to treat relevant subpopulations of cystinosis patients affected by nonsense mutations.^20,69^ Alternatively, the potential use of certain TRIDs also showing NMD decay inhibition as single therapies has also been reported, with Geneticin representing the most relevant example. This aminoglycoside was proven to restore normal levels of the cystinosin transcript by qPCR in various cell lines upon 48 h of treatment while also inducing expression of the protein, detectable by immunoblotting. Nonetheless, the toxicity of Geneticin complicates its use in human patients, which has restricted its use in proof-of-principle studies.^70^

Despite promising results, there remain challenges and hurdles to fully realize the potential benefit of these known drugs for the treatment of cystinosis, not least of which is the complexities involved in drug repurposing.^71^ Further work is needed beyond in vitro experiments, which includes the optimization of pharmacokinetic profiles and the modulation of the pharmacological activity of the discussed scaffolds in order to precisely understand how their action could have a measurable positive impact on the overall disease in humans and lead to the development of truly curative therapeutic approaches in the medium-long-term.

## Conclusions and Perspectives

Despite the efforts exerted over the past few years to develop novel treatments, as of yet, cysteamine is the only clinically validated drug to treat cystinosis. Its known side effects and drawbacks reduce patient compliance, jeopardizing the control of disease progression. In addition to these problems, cysteamine is not a curative treatment. Therefore, there is an urgent need to find novel therapies that can overcome these challenges.

The most straightforward strategy is a delayed-release formulation of cysteamine, which does alleviate some of the problems attributed to the treatment, especially the sleep disruption caused by the short dosing interval. Although several advancements have been made regarding the development of novel cysteamine formulations, the simple optimization of cysteamine administration is insufficient to give a truly effective response to many of the main drawbacks of the treatment, such as halitosis and gastrointestinal side effects, and should only be considered as a complement to other more innovative approaches.

The development of cysteamine prodrugs, especially those that selectively target the lysosome, appears to be the most promising strategy to deliver much-needed improved treatment for cystinosis patients in the short term. Although delayed, recent funding guarantees that clinical trials to assess the CF10 prodrug will proceed in the near future. The field eagerly awaits the results of these trials, as it could lead to the reduction of several of the side effects reported with the traditional treatment, as cysteamine prodrugs can lead to an increase in the half-life of cysteamine and avoid its release prior to it reaching the lysosome, as discussed throughout this review.

Despite the clear promise of cysteamine prodrugs, if they serve only as a precursor to cysteamine, then they cannot be considered a curative treatment. The conscientious study of the pathophysiology of the disease has led to an emerging trend in novel therapies targeting different altered pathways. While promising, this new paradigm is still in its infancy, and many of the molecules suggested in published studies as potential drug candidates may be seen as adjuvants to cysteamine therapy and would be required to be coadministered in any future clinical trials. Small molecules that target general mechanisms, rather than specific biological targets, may suffer from a lack of suitable biomarkers. The advance in these new experimental therapies, which benefit from the growing knowledge of the disease and the improvement of medicinal chemistry techniques, is however crucial to progress to a curative treatment for cystinosis in the medium-long-term.

## §

Department of Chemistry, School of Natural Sciences, University of Manchester, Oxford Road, Manchester M13 9PL, U.K

## Abbreviations

AMP: adenosine monophosphate
AMPK: AMP-activated protein kinase
ATP: adenosine triphosphate
AUC: area under the curve
DMS: dimethylsulfide
EMA: European Medicines Agency
ESRD: end-stage renal disease
FDA: Food and Drug Administration
h: hour
LSD: lysosomal storage disorder
MRC: Medical Research Council
mRNA: messenger ribonucleic acid
MT: methanethiol
mTOR: mechanistic target of rapamycin
mTORC1: mechanistic target of rapamycin complex 1
NMD: nonsense-mediated mRNA decay
PEG: polyethylene glycol
qPCR: quantitative polymerase chain reaction
TEMPO: 2,2,6,6-tetramethylpiperidinyl-1-oxyl
TRID: translational read-through-inducing drug
TRPML1: mucopilin-1
